# Enhanced Electrochemiluminescence Detection of Dopamine Using Antifouling PEDOT-Modified SPEs for Complex Biological Samples

**DOI:** 10.1021/acsmeasuresciau.4c00053

**Published:** 2024-10-04

**Authors:** Tzu-Yu Kao, Chia-Hung Kuo, Yu-Wei Wu, Shyh-Chyang Luo

**Affiliations:** † Department of Materials Science and Engineering, National Taiwan University, No. 1, Sec. 4, Roosevelt Road, Taipei 10617, Taiwan; ‡ Institute of Molecular Biology, Academia Sinica, 128 Academia Road, Section 2, Nankang, Taipei 11529, Taiwan

**Keywords:** electrochemiluminescence, antifouling electrode, functionalized conducting polymer, dopamine detection, screen-printed electrode

## Abstract

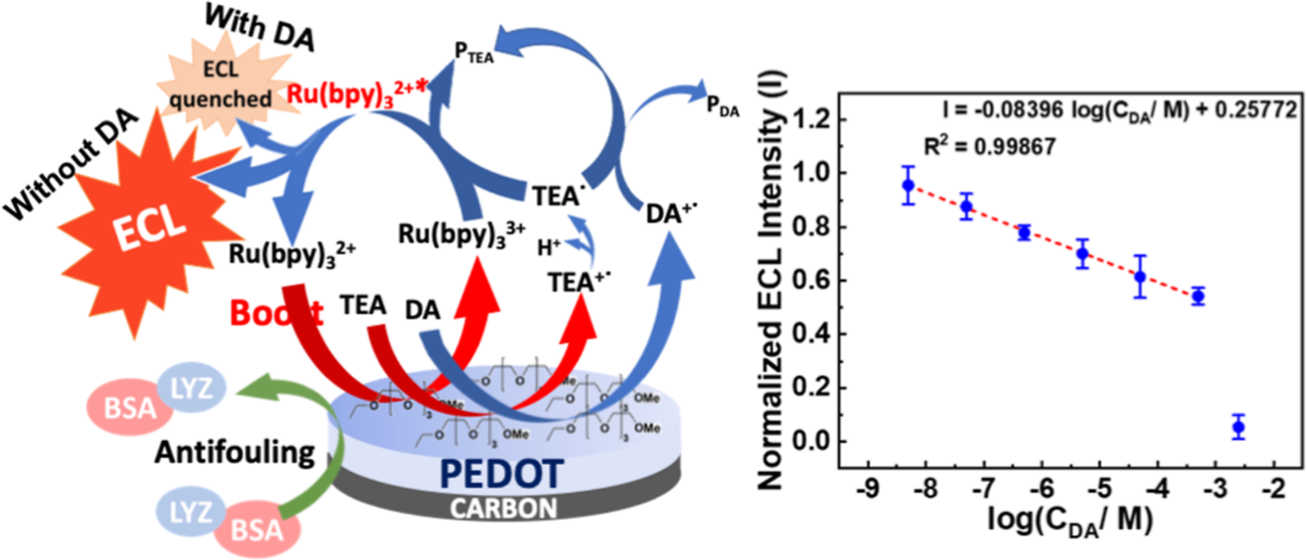

Detecting medically important biomarkers in complex biological samples without prior treatment or extraction poses a major challenge in biomedical analysis. Electrochemical methods, specifically electrochemiluminescence (ECL), show potential due to their high sensitivity, minimal background noise, and straightforward operation. This study investigates the ECL performance of screen-printed electrodes (SPEs) modified with the conductive polymer poly(3,4-ethylenedioxythiophene) (PEDOT) and its derivatives for dopamine (DA) detection. PEDOT modification significantly enhances ECL intensity, improves sensitivity, and expands the linear range for DA detection. Functionalizing PEDOT with ethylene glycol (EG) further enhances stability, specificity, and resistance to interferences for DA detection. These modified SPEs demonstrate the linear range of 1–200 μM and a detection limit as low as 0.887 nM (*S*/*N* = 3), surpassing many previous studies using SPEs. Moreover, the PEDOT-EG_4_-OMe-modified SPEs can reliably detect DA in solutions with high protein concentrations or artificial cerebrospinal fluid. These results suggest that the PEDOT derivative-modified SPE can serve as reusable and sensitive DA sensors in complex biological environments, highlighting the potential of the ECL system for a range of challenging applications.

## 1 Introduction

Dopamine (DA) is a neurotransmitter essential to both cardiovascular and central nervous systems with its levels closely linked to various diseases. Elevated DA can indicate cardiotoxicity, leading to heart issues, while reduced levels are associated with neurological disorders such as Parkinson’s disease, schizophrenia, Alzheimer’s disease, stress, and depression.^1^ Accurate measurement of DA is crucial for diagnosing and managing these conditions. Common analytical techniques include fluorescent sensors^2^ and electrochemical methods.^3,4^ Electrochemical methods are relatively easy to perform but can be interfered with by molecules like uric acid and ascorbic acid,^5^ complicating DA sensing in complex biological samples such as human serum,^6^ urine,^7^ or cerebrospinal fluid.^8,9^ Therefore, the development of specific and low-cost biosensors is highly desirable. Electrochemiluminescence (ECL) is a well-established biological analysis method known for its high sensitivity, rapidity, and straightforward operation. Moreover, unlike fluorescent methods, the ECL technique uses an electric potential rather than light to excite the luminophore, leading to a significantly lower background noise, which is a key advantage in sensor applications. During the ECL process, electron-transfer reactions create intermediate species in an excited state that emit light as they return to the ground state. Among all ECL reagents, Ru(bpy)_3_^2+^ and its derivatives are the most representative and widely used due to their water solubility, high electrochemical stability, and ability to be repeatedly regenerated in ECL reactions.^10^ The ECL reaction of Ru(bpy)_3_^2+^ system has been utilized in various fields, particularly in biosensing, including RNA quantification,^11^ pharmaceutical studies,^12^ and immunoassays.^13^

Recently, many researchers have been working on constructing DA sensors based on ECL. Various chemicals are used as luminophores, such as metal nanoclusters,^14,15^ polycyclic aromatic hydrocarbons,^16^ tris(bipyridine)ruthenium(II) (Ru(bpy)_3_^2+^),^17^ and luminol.^18^ These ECL systems have functioned effectively as DA sensors, achieving detection limits below 1 nM in a phosphate-buffered saline (PBS) solution. Beyond developing ECL luminophores, surface properties, such as impedance, morphology, and nanostructures, significantly influence ECL performance and electrode stability. Materials such as covalent organic frameworks,^19^ nanoparticles,^20,21^ and conducting polymers^22^ are used for surface modification to enhance the ECL signal. Among these, coating electrode surfaces with conducting polymers is one of the most promising approaches.

Conducting polymers such as poly(3,4-ethylenedioxythiophene) (PEDOT) are particularly noteworthy in this context. They exhibit outstanding conductivity due to their conjugated double bonds, facilitating efficient charge transfer across the polymer chain. Besides, PEDOT demonstrates excellent electrochemical and chemical stability, making it highly durable under various operating conditions. Its good biocompatibility further enhances its suitability for electrochemical biosensing applications.^23−26^ When applied as a film on the electrode surface, PEDOT significantly boosts electron-transfer reactions, generating strong ECL signals and improving sensing ability while lowering the detection limit.^15^ This enhancement also enables the construction of an ECL and electrochemical dual-mode DA sensor.

Additionally, the monomer of PEDOT can be functionalized into various derivatives to meet the specific requirements of the biosensors. For instance, functionalizing PEDOT with antifouling groups such as phosphorylcholine (PC)^27,28^ or ethylene glycol (EG)^29^ can significantly reduce nonspecific protein adsorption, which is a common challenge in biosensor applications. The presence of nonspecific proteins on the sensor surface can impede the diffusion of target molecules to the sensing interface, thereby diminishing the overall performance and accuracy of the sensor.^30^ By incorporating antifouling groups, the functionalized PEDOT can maintain a cleaner surface, thus enhancing the sensitivity and accuracy of the biosensor.^31−33^ This approach not only enhances the detection capabilities of biosensors but is also essential for developing high-performance sensing devices that are robust and reliable in complex biological conditions.^34^ Furthermore, certain studies concentrate on DA sensing in actual samples like DA hydrochloride injection, human urine,^35,36^ and blood.^37,38^ These studies demonstrate the capability to detect DA in complex media with detection limits ranging from 100 nM to several μM.

**Scheme 1:**
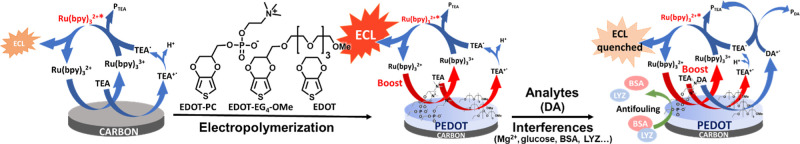
Schematic Illustration of the PEDOT Derivative-Modified Surface Effect on Boosting the ECL Reaction, Demonstrating the Impact of the PEDOT Derivative Film on the Resistance to Interference Molecules for DA Detection

In this study, we address this challenge by investigating the ECL performance in the presence of interfering molecules, aiming to further enhance the detection limit of DA in complex biological samples. As illustrated in Scheme 1, we first synthesize the 3,4-ethylenedioxythiophene (EDOT) monomer with antifouling groups, PC-functionalized EDOT (EDOT-PC), and four EG-functionalized EDOT (EDOT-EG_4_-OMe). Subsequently, we conducted electropolymerization to coat these monomers onto screen-printed electrodes (SPEs) and investigate the ECL behavior of four types of SPEs: bare-SPE, PEDOT-modified SPE, PEDOT–PC-modified SPE, and PEDOT-EG_4_-OMe-modified SPE. PEDOT aims to reduce the surface impedance and accelerate the electron-transfer reaction, thereby enhancing the ECL signal and electrochemical properties. Additionally, PEDOT functionalized with a PC or EG group is intended to enhance the resistance to interferences. The antifouling properties of these PEDOT derivative-modified electrode surfaces were examined to understand their capabilities for DA sensing in complex biological samples.

## 2 Materials and Methods

### 2.1 Materials

Acetonitrile, ammonium peroxodisulfate, ascorbic acid, bovine serum albumin (BSA), calcium chloride, d(+)-glucose, DA hydrochloride, lithium perchloride, magnesium chloride, PBS, potassium chloride, lysozyme (LYZ), minimum essential medium (MEM), sodium bicarbonate, sodium dodecyl sulfate (SDS), sodium chloride, triethylamine, tripropylamine, tris(2,2′-bipyridyl)dichlororuthenium(II) hexahydrate, and uric acid were purchased from Sigma-Aldrich. Potassium ferricyanide was purchased from Acros Organics. EDOT was purchased from the Tokyo Chemical Industry (TCI). Hydroxymethyl-3,4-ethylenedioxythiophene (EDOT-OH) was purchased from Angene Chemical. These chemicals were used without further purification. Besides, EDOT-PC and EDOT-EG_4_-OMe monomers were synthesized by following the previous study.^29,39^

### 2.2 Electropolymerization of PEDOT Derivatives

The electropolymerization was carried out using a potentiostat (PGSTAT128N, Autolab). All experiments were conducted with SPEs (DRP-110, Metrohm DropSens). The electrode system consisted of a flat ceramic card with a circular carbon working electrode (4 mm diameter), a carbon auxiliary electrode, and a silver pseudoreference electrode. The 10 mM EDOT, EDOT-PC, or EDOT-EG_4_-OMe was dissolved in acetonitrile containing 100 mM LiClO_4_, while the EDOT-OH monomer was dissolved in an aqueous solution with 100 mM LiClO_4_ as the supporting electrolyte and 50 mM SDS as a surfactant. All PEDOT derivative films were synthesized on the working electrode of the SPE by applying a cyclic potential from −0.4 to 1.0 V (vs Ag/Ag^+^) at a scan rate of 0.1 V/s for 1 cycle. The PEDOT-modified SPE was coated with a layer of PEDOT, and the PEDOT-PC-modified electrode or the PEDOT-EG_4_-OMe-modified electrode was coated first with PEDOT-OH as an adhesion layer, followed by PEDOT-PC or PEDOT-EG_4_-OMe as an antifouling layer.

### 2.3 Electrochemical Properties

The three-electrode setup consisted of a SPE as the working electrode, Ag/AgCl as the reference electrode, and a wire as the counter electrode in a [Fe(CN)_6_]^3–/4–^ solution. Specifically, the [Fe(CN)_6_]^3–/4–^ solution contained 10 mM K_3_Fe(CN)_6_ and 10 mM K_4_Fe(CN)_6_ in 0.1 M PBS (pH 7.4). For the electrochemical impedance spectroscopy (EIS) experiments, spectra were recorded over the frequency range from 10^–1^ to 10^5^ Hz at a potential of 0.19 V (vs Ag/AgCl). The differential pulse voltammetry (DPV) spectra were recorded over the potential range from −0.2 to 0.6 V.

### 2.4 ECL Measurement

In this research, ECL measurements were performed at room temperature using a SPE and a DRP-SpectroECL instrument (Metrohm DropSens), with a microspectrometer cell as the detector. Data acquisition and analysis were conducted by using the DropView SPELEC software. All experiments were carried out in a 50 μL 0.1 M PBS solution containing 1 mM Ru(bpy)_3_^2+^ as the luminophore and 50 mM triethylamine as the coreactant. The potential was applied in the cyclic voltammetry (CV) mode, ranging from 0.0 to 1.2 V at a scan rate of 0.1 V/s with an integral time of 250 ms.

### 2.5 DA Samples

For the DA analysis, a 10 mM stock solution was prepared in 0.1 M PBS and diluted to the required concentration in 0.1 M PBS daily. A series of solutions containing DA were prepared for DA tests without interference. Starting with stock solutions of 1 nM and increasing concentrations up to 10 mM, each solution was then diluted 1:1 (v/v) in a 2 mM Ru(bpy)_3_^2+^ luminophore solution. This resulted in final DA concentrations of 0.5 nM, 1 nM, 5 nM, 10 nM, 50 nM, 100 nM, 500 nM, 1 μM, 5 μM, 10 μM, 50 μM, 100 μM, 500 μM, 1 mM, 5 mM, 10 mM, 50 μM, 100 mM, 500 μM, 1 mM, and 5 mM.

## 3 Results and Discussion

### 3.1 ECL Performance on Different Electrode Surfaces

**Figure 1:**
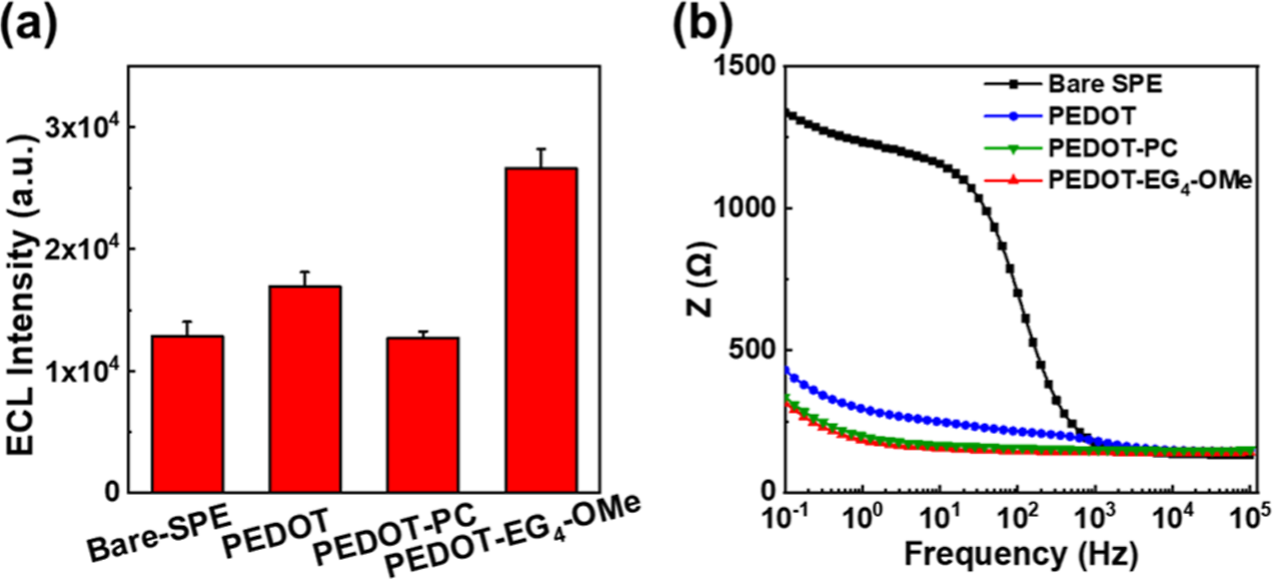
(a) ECL intensity with 1 mM Ru(bpy)_3_^2+^ and 50 mM TEA solution on different electrode surfaces. (b) EIS of PEDOT derivative-modified SPEs compared with the bare-SPE.

First, we measured the ECL intensity of different electrodes, as shown in Figure 1a. The average ECL intensity of the bare-SPE is 12851.7 (a.u.). Meanwhile, the PEDOT-PC-modified SPE showed no significant increase in ECL intensity (12701.0 (a.u.)). The other two SPEs modified with the conducting polymer PEDOT exhibited higher ECL intensities. Specifically, the ECL intensities of the PEDOT-modified and PEDOT-EG_4_-OMe-modified SPE were 16925.7 au and 26583.7 (a.u.), respectively. This indicates that the PEDOT film increased the ECL intensity by 31.7%, and the PEDOT-EG_4_-OMe film increased the ECL intensity by 106.9%. We consider that these effects can be attributed to two main factors. First, electrodes modified with PEDOT derivatives decrease the surface impedance, thereby accelerating the ECL-related oxidative reactions. To support this notion, EIS measurement was conducted to assess the electrochemical properties of PEDOT derivative-modified electrodes, as shown in Figure 1b, with a bare-SPE used for comparison. The results demonstrated that the PEDOT-EG_4_-OMe-modified SPE exhibited the lowest impedance, followed by the PEDOT-PC-modified SPE and PEDOT-modified SPE, all of which showed much lower impedance than the bare-SPE. This suggests that the PEDOT derivatives effectively reduced surface impedance, enhanced electron-transfer reactions, and supported improved ECL performance, consistent with the ECL test results shown in Figure 1a. Second, the wettability of the surface also plays a significant role. As illustrated in Figure S2, PEDOT-EG_4_-OMe is more hydrophilic than the PEDOT-modified SPE and the bare-SPE. This increased hydrophilicity allows for greater access of coreactant and luminophore molecules to the surface, enhancing the ECL signal. However, PEDOT-PC did not show improved ECL behavior despite being more hydrophilic than PEDOT-EG_4_-OMe. This could be attributed to the charged choline and phosphatidic acid groups, which requires further investigation.

### 3.2 ECL Stability

**Figure 2:**
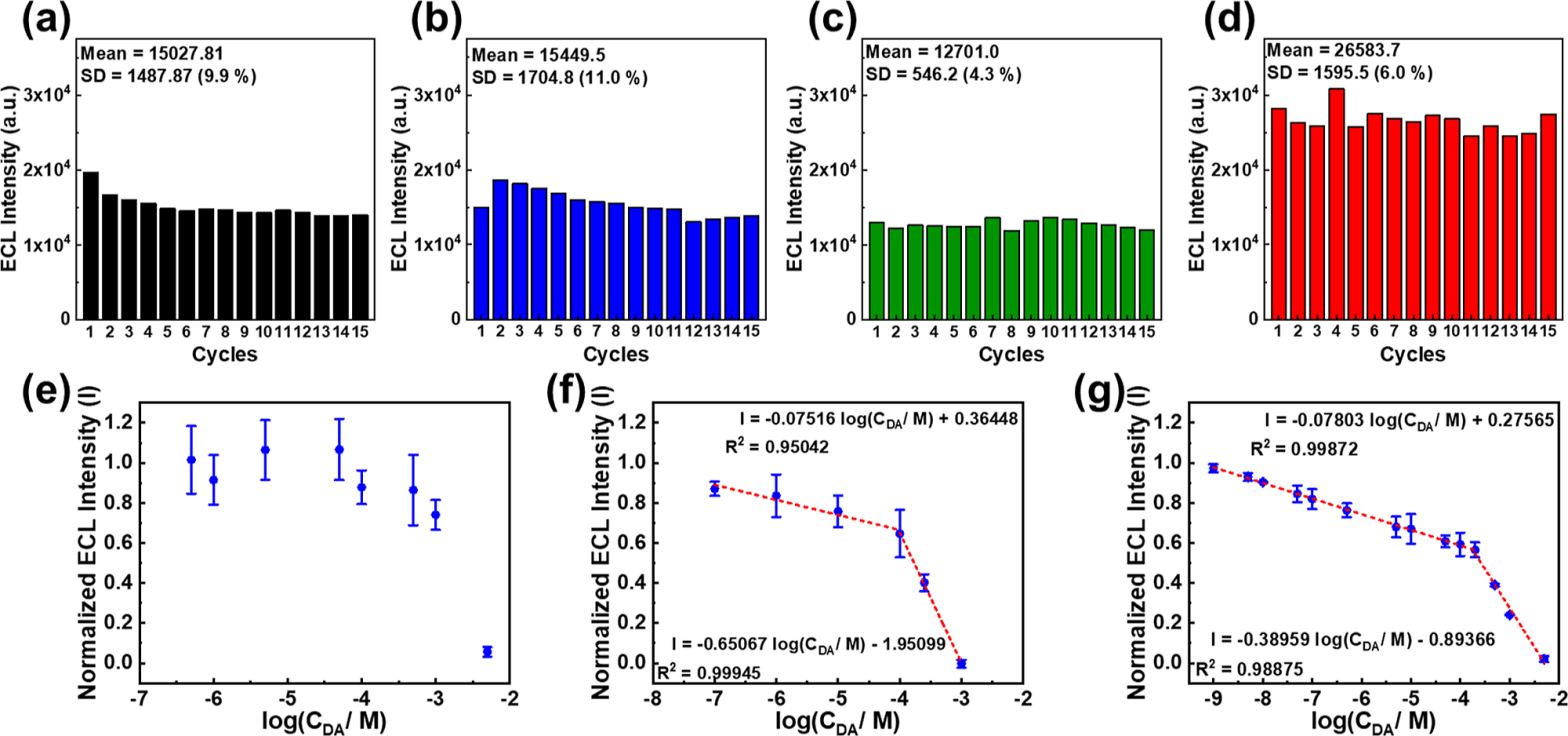
ECL intensity variance in a 15-time test with 1 mM Ru(bpy)_3_^2+^ and 50 mM TEA solutions on (a) bare-SPE, (b) PEDOT-modified-SPE, (c) PEDOT-PC-modified-SPE, and (d) PEDOT-EG_4_-OMe-modified-SPE. Calibration plot for DA detection without interference on (e) bare-SPE, (f) PEDOT-modified-SPE, and (g) PEDOT-EG_4_-OMe-modified-SPE. Experimental conditions: scanning range, from 0.0 to 1.2 V; scan rate, 0.1 V/s; and integral time, 250 ms.

For biosensors, the ECL stability is a crucial issue. In our tests, we measured the ECL intensities of each electrode 15 times. Since the working solution volume is only 50 μL, the concentration of TEA molecules may change if we conduct many experiments without changing the solution. To maintain consistency, we rinsed the electrode surface with DI water, dried it with nitrogen gas, and loaded a new working solution onto the electrode surface for each ECL test. As shown in Figure 2, the ECL intensity of both the bare-SPE and PEDOT-modified SPE electrodes decreases with the number of tests. The average ECL intensity of the bare-SPE is 15027.81 au with a standard deviation of 9.9%, while the average ECL intensity of the PEDOT-modified SPE is 15449.47 au with a standard deviation of 11.0%. On the other hand, the ECL intensity of the PEDOT-PC-modified SPE is 12701.0 (a.u.) with a standard deviation of about 4.3%, and the ECL intensity of the PEDOT-EG_4_-OMe-modified SPE is 25683.65 (a.u.) with a standard deviation of about 6.6%. The CV plots for the four electrodes during the ECL tests are also presented in Figure S4.

We also examine the surface morphology before and after 15 ECL tests of the PEDOT-EG_4_-OMe-modified SPE by scanning electron microscopy (Figure S5), and there is no obvious difference in the surface morphology, indicating its high stability. While the PEDOT-PC-modified SPE shows the lowest standard deviation in ECL, it did not significantly enhance the ECL intensity compared with the bare-SPE.

Additionally, we conducted optimization experiments to determine how the amount of PEDOT-EG_4_-OMe on the electrode surface and the maximum applied potential influence the ECL intensity. First, we investigated the effect of varying amounts of PEDOT-EG_4_-OMe on the ECL intensity (Figure S6a). The amount of PEDOT-EG_4_-OMe was controlled by adjusting the number of electropolymerization cycles. The results indicate that the thickness of PEDOT-EG_4_-OMe does not significantly affect the ECL intensity. Specifically, compared to the surface electropolymerized for one cycle, the ECL intensity increased slightly by 3.0% for three cycles and 1.4% for ten cycles. Next, we assessed the ECL intensity at different maximum oxidizing potentials (Figure S6b). The results show that applying 1.2 V as the maximum potential yielded the highest ECL intensity. Consequently, we decided to electropolymerize PEDOT-EG_4_-OMe for one cycle as the antifouling layer and to use 1.2 V as the maximum potential for subsequent experiments.

### 3.3 DA Sensing without Interferences

We prepared DA solutions with varying concentrations in PBS buffer to evaluate the DA detection capability of the three electrodes without interference and observed the ECL intensity. In a DA solution, DA undergoes oxidation at the modified surface during an anodic sweep (Figure S7). The resulting oxidation product (DA^+˙^) interacts with the TEA radical, leading to a decrease in the ECL emission. By leveraging the efficient ECL behavior of the Ru(bpy)_3_^2^+^^/TEA system, a straightforward “signal-off” strategy can be proposed for DA detection based on the consumption of the electro-oxidation product of coreactant.^2^ DA quenched the ECL emission on the bare-SPE, and the ECL intensity was decreased gradually with the increase in DA concentration (Figure 2e). However, we could not clearly discern DA samples with concentrations lower than 500 μM by the bare-SPE. In contrast, the PEDOT-modified SPE exhibited a linear relationship between ECL intensity and DA concentration after logarithmic transformation in two linear ranges: 100 nM–100 and 100 μM–1 mM, respectively (Figure 2f). The detection limit was estimated to be 179.9 nM (*S*/*N* = 3). Finally, we found that the detection capability of the PEDOT-EG_4_-OMe-modified SPE was even better than that of the PEDOT-modified SPE. It also exhibited a linear relationship between ECL intensity and DA concentration in two linear ranges: 1 nM–200 μM and 200 μM–5 mM, with a limit of detection estimated to be 0.887 nM (Figure 2g). The coating of the PEDOT-EG_4_-OMe film decreased the detection limit and significantly expanded the linear range significantly. Additionally, we have compared the linear range and detection limit of our sensor with other works. As shown in Table 1, we compare various sensor platforms that detect DA by using different techniques. Furthermore, Table S1 provides a comparison of studies that utilize Ru(bpy)_3_^2^+^^ as a luminophore in ECL-based detection systems.

**Table 1:** Comparison of Analytical Techniques for DA Detection

technique	linear range	LOD	published year	reference
fluorescence	0.5–3 μM	15 nM	2024	(40)
CV	6.25–200 μM	190 nM	2024	(41)
DPV	0.1–10 μM	30 nM	2023	(42)
amperometry	1–170 μM	20 nM	2024	(43)
ECL	1 nM–200 μM	0.887 nM		This work

### 3.4 Selectivity of ECL for DA Sensing

**Figure 3:**
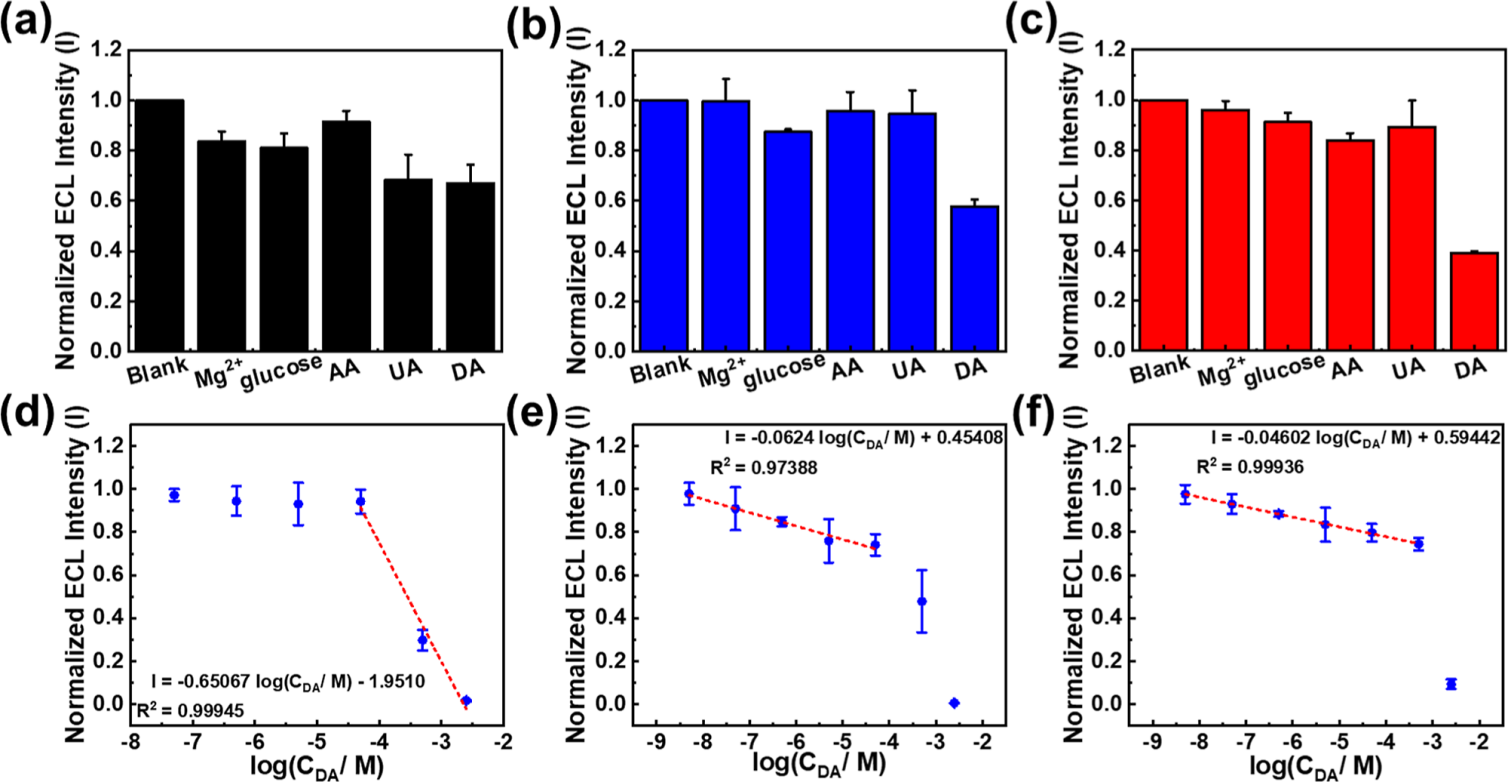
Selectivity of (a) bare-SPE, (b) PEDOT-modified SPE, and (c) PEDOT-EG_4_-OMe-modified SPE with different interferences. Calibration plot for DA detection in MEM solution on (d) bare-SPE, (e) PEDOT-modified SPE, and (f) PEDOT-EG_4_-OMe-modified SPE.

The specificity was investigated with UA, AA, Mg^2+^, and glucose as the interferents. We measured the ECL emission of the Ru(bpy)_3_^2+^/TEA system in the presence of AA and UA at 0.5 mM, glucose, and Mg^2+^ at 5 mM on three electrodes. The ECL emission at the bare-SPE decreased by 33.0% toward 0.5 mM DA. Additionally, the ECL emission decreased by 16.4% in the presence of Mg^2+^ and by 19.1% in the presence of glucose, respectively, and by 8.6% and 31.7% in the presence of AA and UA (Figure 3a). These results indicate that the ECL emission on the bare-SPE was affected by the presence of these molecules, making it challenging to distinguish DA from the other molecules. In contrast, the ECL emission on the PEDOT-modified SPE was decreased by 42.3% in the presence of 0.5 mM DA. However, the ECL emission only reduced by 0.5%, 12.6%, 5.4%, and 4.3% in the presence of Mg^2+^, glucose, AA, and UA, respectively (Figure 3b). Additionally, the ECL emission of the PEDOT-EG_4_-OMe-modified SPE was remarkably decreased toward 0.5 mM DA (61.0%) but remained almost unaltered in the presence of interference molecules. The ECL emission only decreased by 16.1% and 10.7% in the presence of Mg^2+^, glucose, AA, and UA, compared to the ECL emission of the system without interfering species (Figure 3c). These results suggest that PEDOT and PEDOT-EG_4_-OMe improve the selectivity of the SPE, exhibiting a more excellent selectivity for DA over other interfering molecules compared to the bare-SPE.

Moreover, we conducted the recovery test of DA detection under the existence of 0.5 mM AA and UA to validate the accuracy and reliability of the PEDOT-EG_4_-OMe-modified SPE toward DA detection. As Table 2 illustrates, the recovery rates for 0.1, 1, and 10 μM DA are 105.6%, 101.5%, and 104.0%, respectively. Besides, the relative standard deviations are 5.30%, 6.11%, and 3.49%, respectively. These results indicate that the PEDOT-EG_4_-OMe modified SPE exhibits considerable accuracy and reliability.

**Table 2:** Recovery Test Results in the Presence of Interferences

environment	DA added (μM)	found (μM)	recovery (%)	RSD (%, *n* = 5)
0.5 mM AA and UA in PBS	0.1 μM	0.1056 μM	105.6	5.30
	1 μM	1.015 μM	101.5	6.11
	10 μM	10.40 μM	104.0	3.49

### 3.5 DA Sensing with General Interferences

After confirming the selectivity of the three electrodes toward DA, we attempted to detect DA molecules in a MEM solution, which contains 13 amino acids, 8 vitamins, cations (K^+^, Na^+^, and Mg^2+^), anions (CO_3_^2–^ and PO_4_^3–^), and glucose. As shown in Figure 3d–f, the ECL emission of the Ru(bpy)_3_^2+^/TEA system on the bare-SPE was quenched by DA with the ECL intensity decreasing as the DA concentration increased from 50 μM to 2.5 mM. However, it was challenging to clearly discern DA samples with concentrations lower than 50 μM using the bare-SPE. In contrast, the PEDOT-modified SPE and PEDOT-EG_4_-OMe-modified SPE exhibited a good linear relationship between ECL intensity and DA concentration after logarithmic transformation in the ranges of 5 nM–50 μM and 5 nM–2.5 mM, respectively. The limit of detection for the PEDOT-modified SPE and PEDOT-EG_4_-OMe-modified SPE was estimated to be 121.2 and 1.403 nM, respectively. These detection limits are nearly the same as those achieved when detecting DA molecules without interferences, indicating that the conducting polymer films have a higher resistance to small-molecule interferences. These detection limits are nearly the same as those achieved when detecting DA molecules without interferences, indicating that the conducting polymer films have a higher resistance to interferences. While the detection limit and linear range remained unchanged, the sensitivity of DA sensing dropped by approximately 40% for both the PEDOT-modified SPE and PEDOT-EG_4_-OMe-modified SPE. For the PEDOT-modified SPE electrode, the sensitivity decreased from 1397.3 (a.u./logC_DA_) to 896.6 (a.u./logC_DA_). Similarly, for the PEDOT-EG_4_-OMe-modified SPE, the sensitivity decreased from 2532.2 (a.u./logC_DA_) to 1488.48 (a.u./logC_DA_). This reduction in sensitivity is attributed to small-molecule interferences that reduce the likelihood of electron-transfer reactions between DA and TEA radicals, thereby weakening the ECL quenching ability of the DA molecules.

### 3.6 ECL in the Presence of Protein Interferences

After confirming the resistance of the PEDOT-modified SPE and PEDOT-EG_4_-OMe-modified SPE to small-molecule interferences, we investigated their ability to resist fouling by macromolecular interferences, particularly protein molecules. BSA and LYZ were chosen as model proteins for this study. We measured the ECL intensity after the protein-incubated electrode surface was rinsed with DI water.

**Figure 4:**
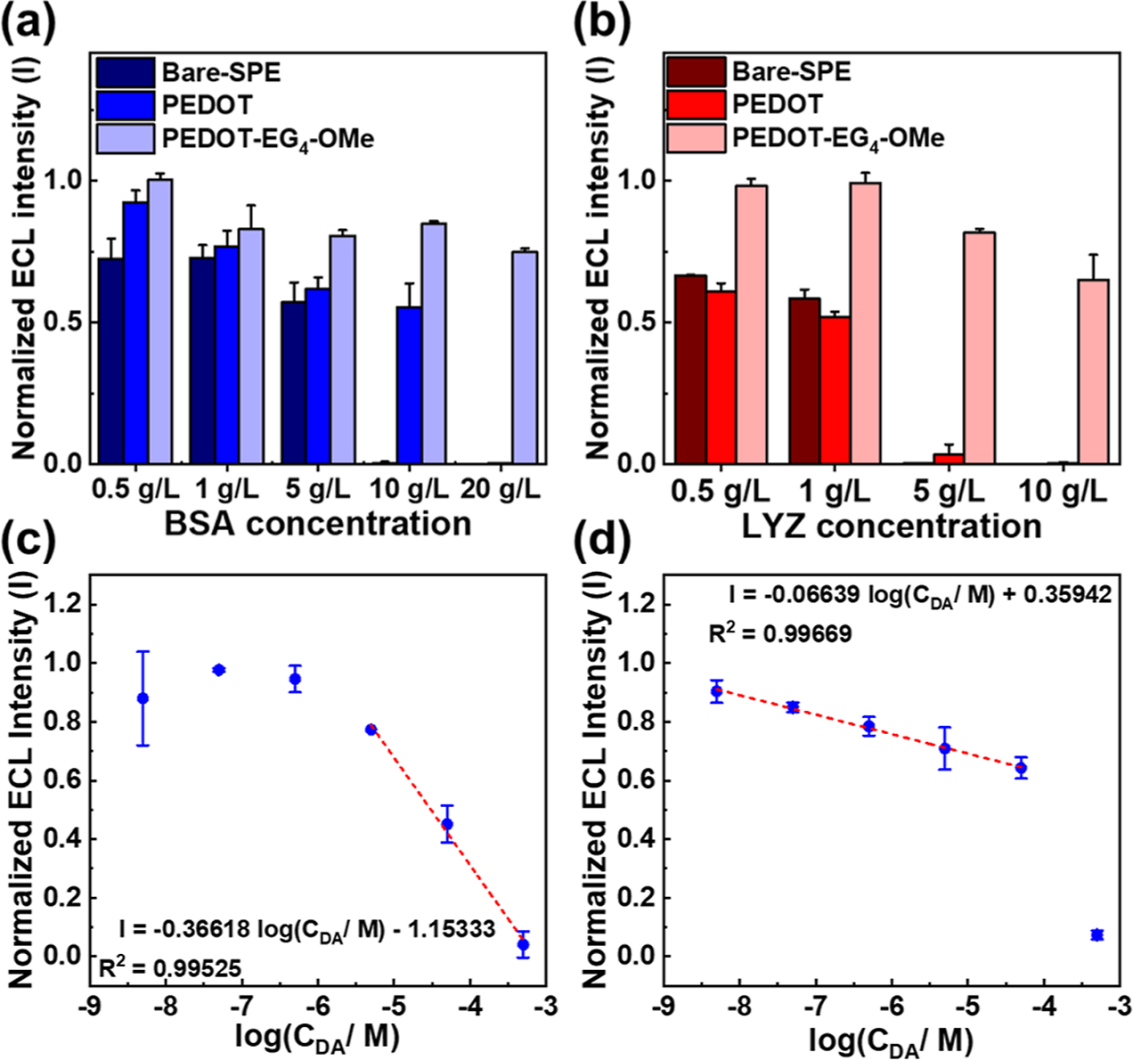
ECL intensity variance of different electrode surfaces after incubation in (a) BSA protein solution and (b) LYZ protein solution. Calibration plot for DA detection in protein solution (0.5 g/L BSA and 0.5 g/L LYZ) on (a) PEDOT-modified SPE and (b) PEDOT-EG_4_-OMe-modified SPE.

First, we investigated the ECL behavior after BSA incubation. As shown in Figure 4a, the ECL intensity of the bare-SPE decreased rapidly with increasing BSA concentration in the incubation solutions. After incubation with 0.5 g/L BSA, the ECL intensity decreased to 72.6%. The bare-SPE completely lost its ability to generate an ECL signal after incubation with 10 g/L BSA. In contrast, the PEDOT-modified SPE maintained a higher ECL intensity (92.4%) after incubation with 0.5 g/L BSA and could tolerate up to 20 g/L BSA concentration, which is twice the tolerance of the bare-SPE. Similarly, the PEDOT-EG_4_-OMe-modified SPE maintained a higher ECL emission (99.7%) with 0.5 g/L BSA and maintained 74.9% ECL emission even after incubation with 20 g/L BSA compared to the ECL emission before BSA incubation. Second, we examined the ECL behavior following LYZ incubation. As illustrated in Figure 4b, the ECL intensity of both bare-SPE and PEDOT-modified SPE decreased significantly after incubation with 0.5 g/L LYZ, with the ECL intensity decreasing to 66.6% and 61.0%, respectively. When the LYZ concentration increased to 5 g/L, both the bare-SPE and PEDOT-modified SPE lost their ability to generate ECL signals. In contrast, the PEDOT-EG_4_-OMe-modified SPE exhibited distinctive antifouling properties, maintained 98.2% ECL emission with 0.5 g/L LYZ, and maintained 81.6% ECL emission even after incubation with 5 g/L LYZ, compared to the ECL emission before LYZ incubation. We also conducted DPV to assess the antifouling properties of the surfaces (Figure S8). Our findings revealed that while the PEDOT-modified SPE shows slightly better resistance to protein molecules than bare-SPE, both electrodes exhibit limited antifouling properties against proteins. Only the PEDOT-EG_4_-OMe-modified SPE demonstrates distinct antifouling properties against both BSA and LYZ, consistent with the results of the ECL test.

### 3.7 DA Sensing with Protein Interferences

In the previous section, we established that the PEDOT-EG_4_-OMe-modified SPE incubated with protein solutions can be reused after rinsing with DI water. We then proceeded to detect DA samples in the presence of protein solutions. DA solutions were prepared in PBS buffer, with BSA and LYZ proteins added at a concentration of 0.5 g/L to serve as interferences. Notably, the PEDOT-modified SPE electrode lost its ability to detect DA concentrations below 5 μM (Figure 4c). In contrast, the PEDOT-EG_4_-OMe-modified SPE’s detection capability remained unaffected under these conditions, demonstrating a linear relationship between ECL intensity and DA concentration in the range of 5 nM–50 μM, with an estimated detection limit of 0.989 nM (Figure 4d). Additionally, unlike the detection of DA in MEM solution, where numerous interfering molecules significantly influence detection sensitivity, the sensitivity in the protein solution decreased by only 19.2%, from 2532.2 (a.u./logC_DA_) to 2046.8 (a.u./logC_DA_), indicating that the sensitivity remained relatively stable.

### 3.8 DA Sensing in Artificial Cerebrospinal Fluid

To simulate the real application of the DA sensor, we attempted to detect DA samples in artificial cerebrospinal fluid (aCSF). DA solutions were prepared in our self-prepared aCSF, which contains various ions including 125 mM sodium chloride (NaCl), 2.5 mM potassium chloride (KCl), 1.25 mM sodium dihydrogen phosphate (NaH_2_PO_4_), 25 mM sodium bicarbonate (NaHCO_3_), 15 mM glucose, 2 mM calcium chloride (CaCl_2_), and 2 mM magnesium chloride (MgCl_2_). First, we observed the ECL intensity in aCSF and compared it with solutions containing different interferences.

**Figure 5:**
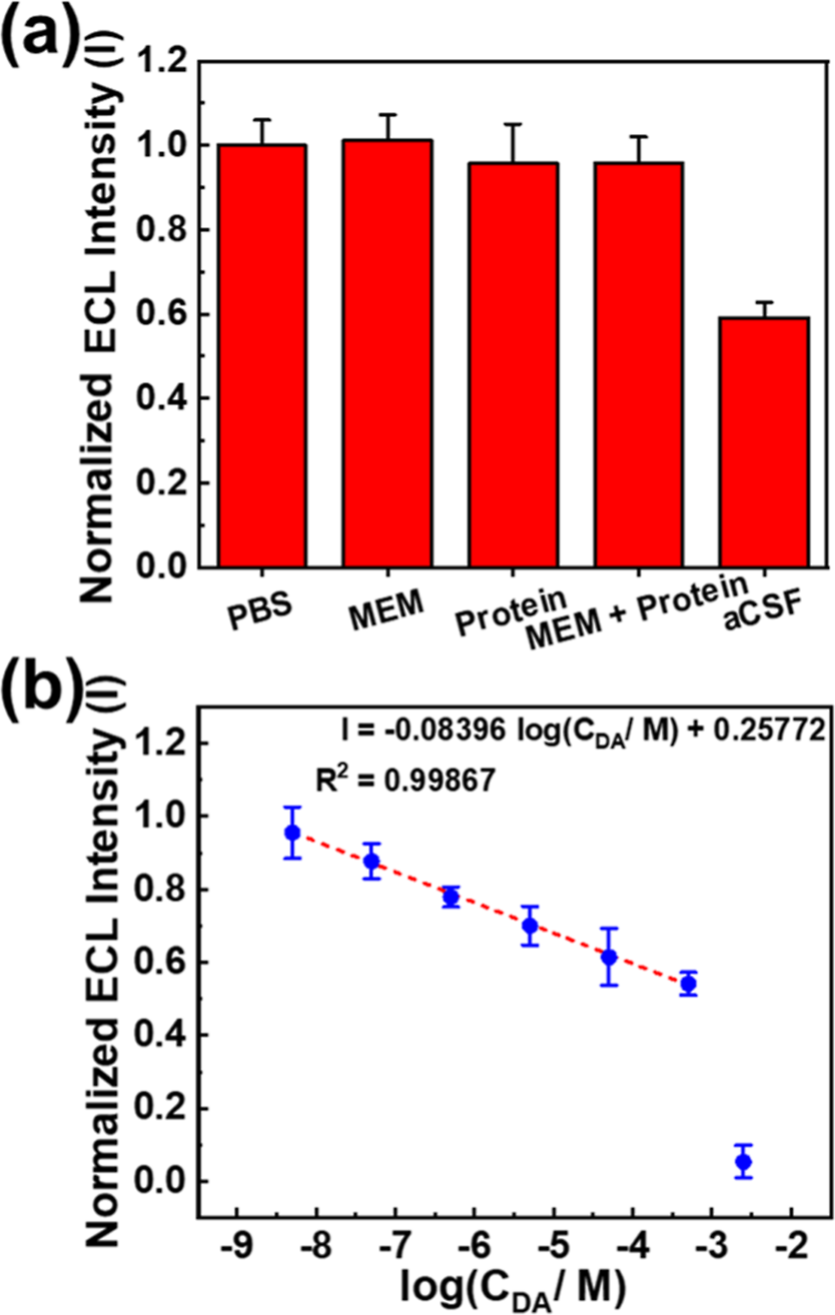
(a) ECL intensity variance of the PEDOT-EG_4_-OMe-modified SPE in different solutions. (b) Calibration plot for DA detection in aCSF on the PEDOT-EG_4_-OMe-modified SPE.

As shown in Figure 5a, while the ECL intensity in MEM solution, protein solution containing 0.5 g/L BSA and LYZ, and the mixture of MEM solution and protein solution only slightly change +1.1%, −4.3%, and −4.2%, respectively, the ECL intensity of the PEDOT-EG_4_-OMe-modified SPE decreased significantly (40.7%) compared to the ECL intensity in PBS. This decrease can be attributed to the high concentration of calcium ions, which easily precipitate with phosphate anions, hindering the electron-transfer process necessary to generate the ECL signal. Despite the significant drop in ECL intensity in aCSF, the PEDOT-EG_4_-OMe-modified SPE’s detection capability toward DA remained unaffected under these conditions. It demonstrated a linear relationship between ECL intensity and DA concentration in the range of 5 nM–500 μM, with an estimated detection limit of 3.119 nM (Figure 5b). Additionally, the wavelength vs intensity plot is presented in Figure S10. By constructing a calibration curve between the normalized ECL intensity and the logarithm of DA concentration, we can estimate the DA concentration in real samples.

## 4 Conclusions

This study used the Ru(bpy)_3_^2+^/TEA ECL system on a SPE for DA detection. Modifying the SPEs with a layer of the conductive polymer PEDOT significantly enhanced the ECL intensity, improving sensitivity and the linear range for DA detection. Furthermore, the PEDOT-EG_4_-OMe film enhanced the stability and specificity for DA detection, reducing the impact of common small-molecule interferents. This electrode can detect DA molecules in the presence of various interferences, achieving a detection limit below 1 nM, surpassing most studies using SPEs. In summary, this study successfully transformed commercially available SPEs into reusable DA sensors suitable for operation in complex environments, expanding the applicability of the ECL system across various challenging conditions.

## Supporting Information Available

Additional experimental details, methods; supplementary analysis results including contact angle, SEM, and DPV; comparison with other works (PDF)
