# Supplementary material for: Enhanced Electrochemiluminescence Detection of Dopamine Using Antifouling PEDOT-Modified SPEs for Complex Biological Samples
Source: ACS Meas Sci Au. 2024 Oct 4;4(6):712–20. doi: 10.1021/acsmeasuresciau.4c00053 (PMC11659998; doi:10.1021/acsmeasuresciau.4c00053)
Supplement: Supplementary file 1 — tg4c00053_si_001.pdf [file tg4c00053_si_001.pdf]

Supporting Information

Enhanced Electrochemiluminescence Detection of Dopamine Using Antifouling PEDOT-Modified SPEs for Complex Biological Samples

Tzu-Yu Kao,[1] Chia-Hung Kuo,[1] Yu-Wei Wu,[2] and Shyh-Chyang Luo[*, 1]

[1]Department of Materials Science and Engineering, National Taiwan University, No. 1, Sec. 4, Roosevelt Road, Taipei 10617, Taiwan.

[2]Institute of Molecular Biology, Academia Sinica, 128 Academia Road, Section 2, Nankang, Taipei115, Taiwan.

Table of Contents

Synthesis of EDOT-EG_4-OMe monomer……………………………………………………3

ECL performance with different coreactant……………………………………..…………3

Contact angle measurement…………………………….………………………...…4

Cyclic voltammetry plots of four PEDOT derivative modified SPEs………...………………..6

SEM image of PEDOT-EG_4-OMe-modified electrodes surface ……………………..……..………………7

Optimization procedure……………………………………………………………………….7

Cyclic voltammetry plot for dopamine solution ……………………………………………….………8

DPV analysis for protein adsorption ……………………………..……………………………..…………9

Calculation of detection limits……………………………..…………………………………..………10

Dopamine detection in the presence of both macromolecular and small molecule interferences……………………………..…………………………...……..……10

Wavelength vs intensity plot……………………………………………………..………11

Comparison of ECL dopamine sensing ability in real samples with other work ……………12

References ……………………………………………………………………………14

Synthesis of EDOT-EG_4-OMe monomer.

To summarize, NaH (25 mmol, 5 equiv), 18-crown-6 (1 mmol, 0.2 equiv), and EDOT-OH (5 mmol, 1 equiv) were combined and dissolved in dry THF (20 ml). Then, a solution of methyl-PEG_4-bromide (5.5 mmol, 1.1 equiv) was added. The reaction mixture was stirred at room temperature for 24 hours under nitrogen. The mixture was then extracted with CH_2Cl_2, and the organic layer was dried over $MgSO_4$. The solvent was removed under reduced pressure. The crude product was purified by column chromatography, yielding EDOT-EG_4-OMe as a colorless oil.

ECL performance with different co-reactant

The experimental results are shown in **Figure S1.** From the spectrum in **Figure S1(a)**, it can be seen that the ECL systems with all three coreactants detect the highest signal at a wavelength of 620 nm. Among these, ammonium peroxodisulfate ($NH_4S_2O_8$) has the least enhancement effect on ECL, while TEA has the best effect. The maximum ECL intensities for the Bare-SPE with persulfate ions, TEA, and TPrA coreactants at a concentration of 50 mM are 408.4 a.u., 12851.7 a.u., and 1499.9 a.u., respectively. In comparison, the ECL intensities for the PEDOT-modified SPE with persulfate ions, TEA, and TPrA coreactants at the same concentration are 771.4 a.u., 16925.67 a.u., and 3294.25 a.u., respectively. These results indicate that the PEDOT-modified SPE exhibits significantly enhanced ECL performance across all three coreactant systems. The enhancement can be attributed to the improved electron transfer efficiency and increased surface area provided by the PEDOT modification, which facilitates better interaction with the coreactants. In particular, the TEA system shows the highest ECL intensity for both Bare-SPE and PEDOT-modified SPE, suggesting that TEA is the most effective coreactant among those tested.

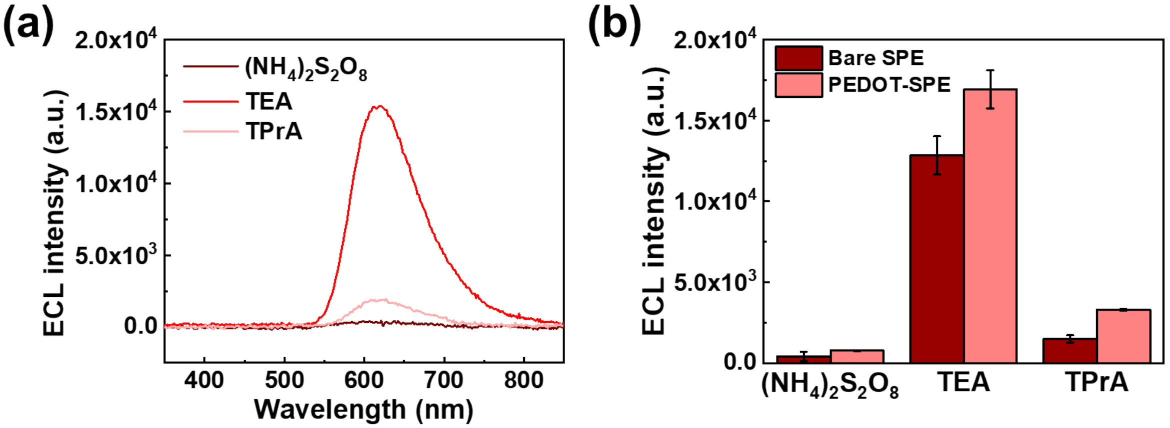

Figure S1. (a) Spectrum of $Ru(bpy)_3^{2+}$ ECL system with different coreactant (50 mM) on PEDOT-modified SPE. (b) The comparison of ECL intensity on different electrode surface with different coreactant.

Contact angle measurement

After completing the surface modification of the electrodes, we conducted contact angle tests to observe the changes in the hydrophilicity of the electrode surfaces. Static contact angles were measured at room temperature using a contact angle goniometer (Sindatek, Taiwan). Droplets of 1.0 μL deionized water were carefully placed on the surface of the samples, and the contact angles were recorded to evaluate the surface hydrophilicity. For the investigation of the surface morphology of the PEDOT-modified screen-printed electrodes, scanning electron microscopy (JEOL JSM-7800F Prime FEG-SEM) was utilized, providing insights into the characteristics and modifications by the PEDOT coating.

As shown in **Figures S2** and **S3**, the contact angles on the SPE surfaces decreased to varying degrees after the electrochemical polymerization of PEDOT derivatives, indicating that the electrode surfaces became more hydrophilic. Specifically, the water-in-air contact angle slightly decreased from 111.7° on the Bare-SPE to 106.6° on the PEDOT-modified SPE. Furthermore, the contact angles of the PEDOT-PC-modified SPE and PEDOT-EG_4-OMe-modified SPE significantly decreased to 9.7° and 50.9°, respectively, demonstrating their increased hydrophilicity.

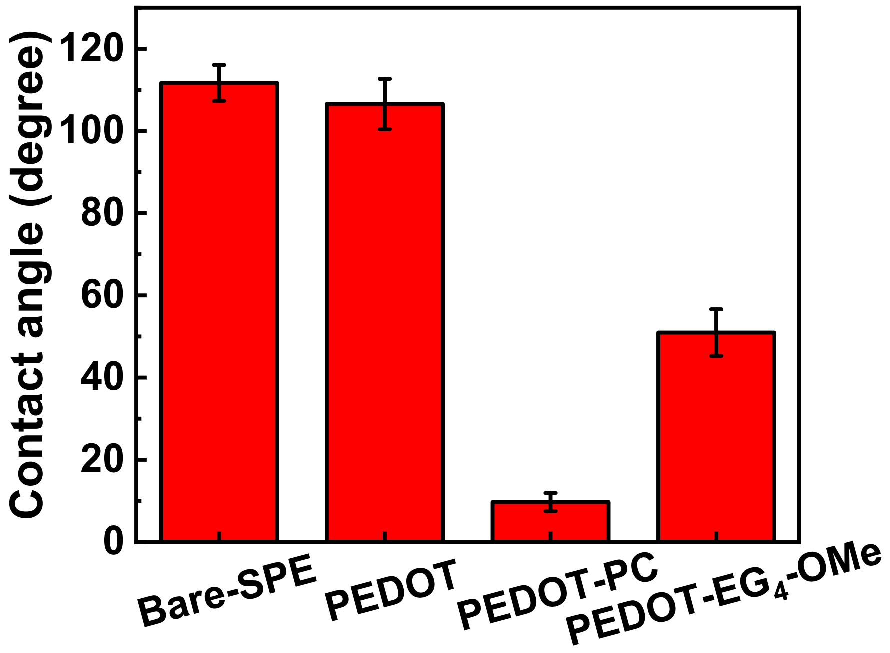

Figure S2. Water-in-air contact angle of different electrode surfaces.

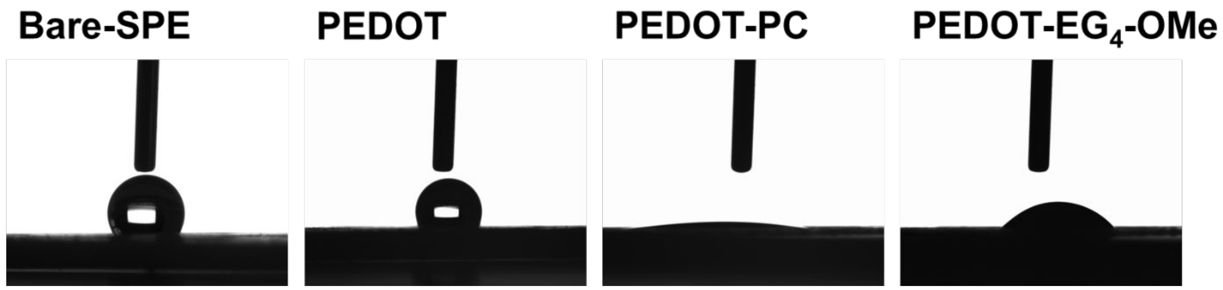

Figure S3. The image of water-in-air contact angle of different surfaces.

Cyclic voltammetry plots of four PEDOT derivative modified SPEs

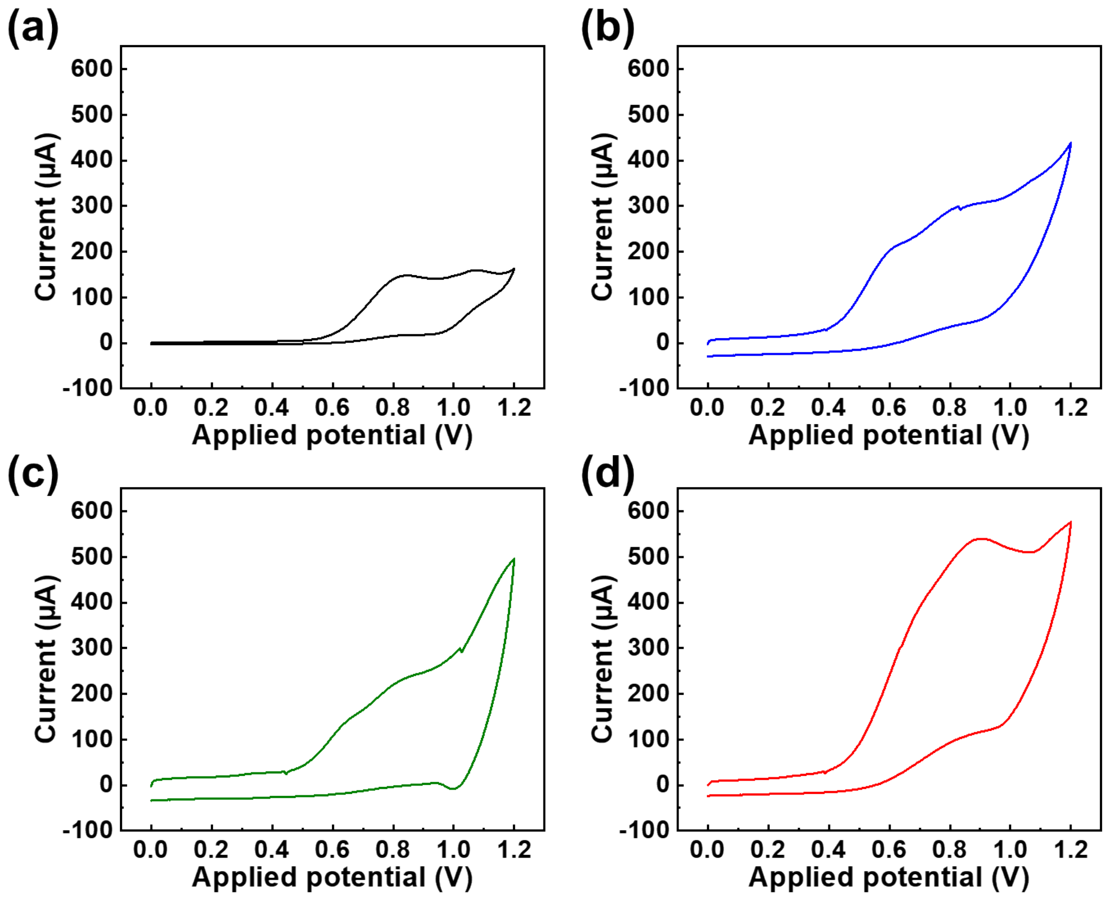

Figure S4. Cyclic voltammetry plot of different surface during ECL test in 1 mM $Ru(bpy)_3^{2+}$ and 50 mM TEA: (a) Bare-SPE (b) PEDOT modified SPE (c) PEDOT-PC modified SPE (d) PEDOT-EG_4-OMe modified SPE.

SEM image of PEDOT-EG_4-OMe-modified electrodes surface

To confirm the changes in the electrode surface morphology before and after the ECL tests, we used a scanning electron microscope to observe the surface of the PEDOT-EG_4-OMe-modified SPE before and after 15 ECL tests. As shown in **Figure S5**, after 15 ECL tests, the electrode surface became slightly rougher, but most of the conductive polymer film remained intact and continued to adhere to the electrode surface.

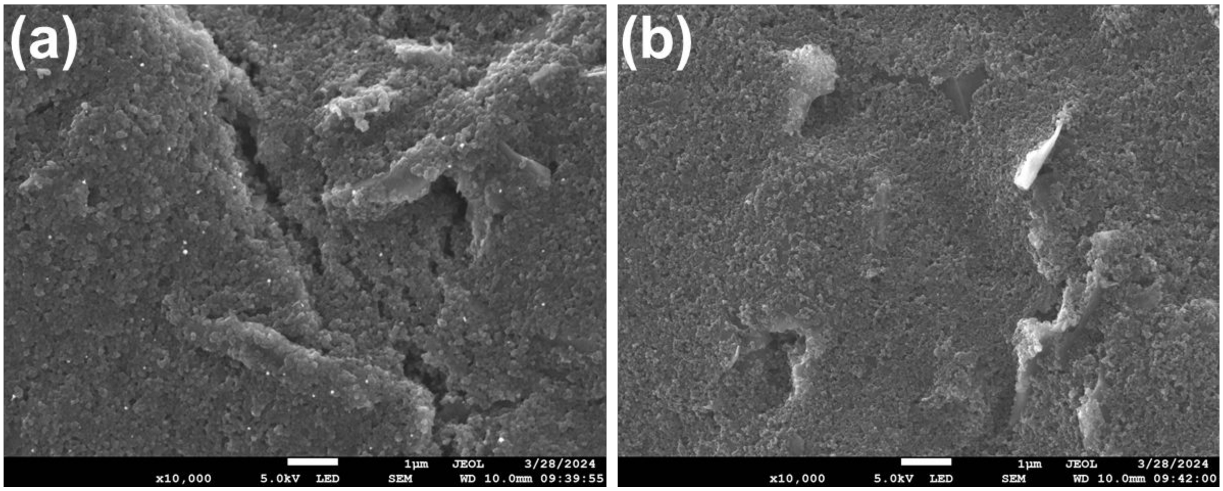

Figure S5. SEM image of PEDOT-EG_4-OMe-modified SPE surface (a) before 15 cycles of ECL test. (b) after 15 cycles of the ECL test.

Optimization procedure

To further optimize the condition when conducting surface modification and ECL test, we investigate the influence on ECL intensity with the amount of PEDOT-EG_4-OMe on the electrode surface (**Figure S6(a)**), the amount of PEDOT-EG_4-OMe was controlled by manipulating the times when conducting electropolymerization. We also tested the ECL intensity when applying different maximum oxidizing potentials (**Figure S6(b)**).

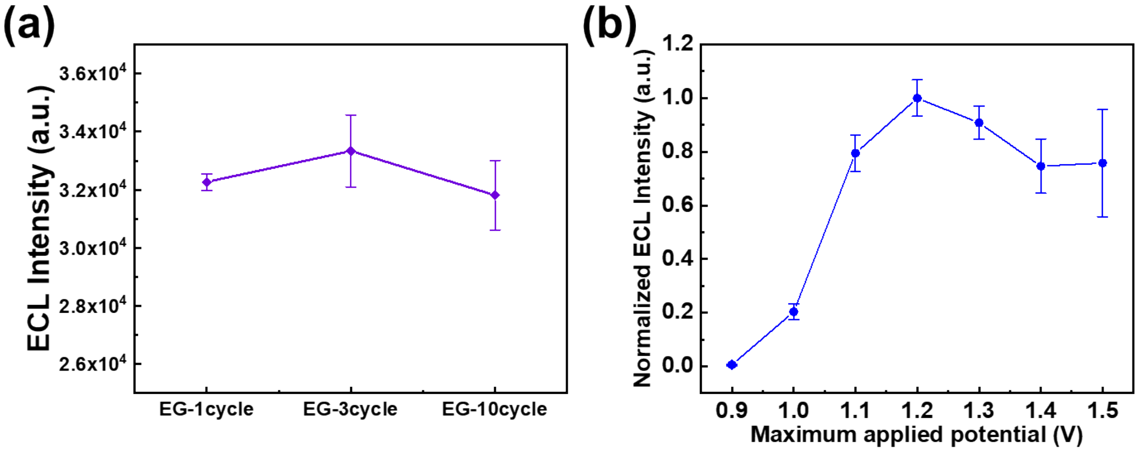

Figure S6. (a) Comparison of ECL intensity as a function of varying amounts of PEDOT-EG_4-OMe. (b) ECL intensity in response to changes in the maximum applied potential.

Cyclic voltammetry plot for dopamine solution.

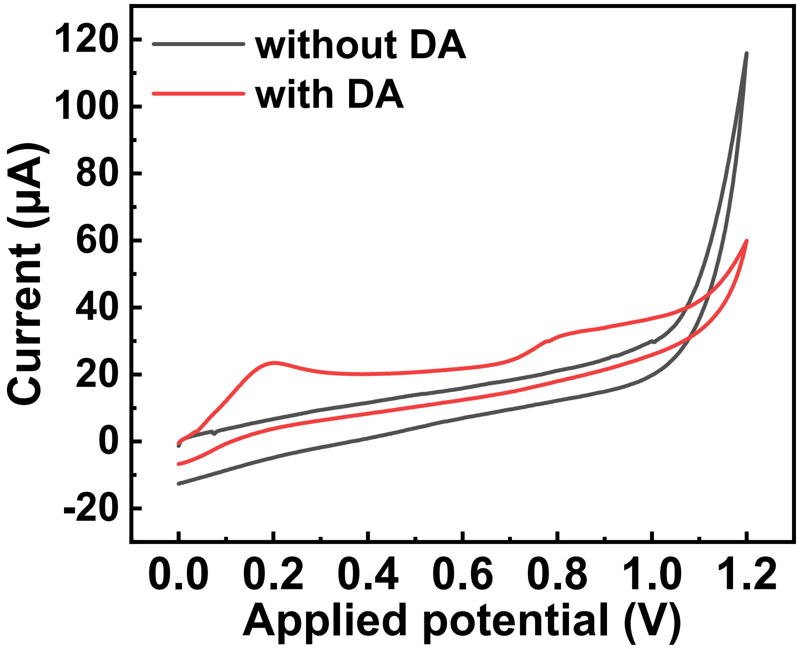

Figure S7. Comparison of cyclic voltammetry plot of PEDOT-EG_4-OMe modified SPE with the existence of dopamine (10 μM) in the solution.

DPV analysis for protein adsorption

Additionally, we performed DPV tests on three types of SPEs both before and after soaking in protein solutions and after rinsing with deionized water. We measured the DPV peak values of the electrodes before and after exposure to 10 g/L BSA or 5 g/L LYZ. As shown in **Figure S8(a)**, for the Bare-SPE, the DPV peak value decreased to 26.36% of its original value after soaking in 10 g/L BSA and to 16.59% of its original value after soaking in 5 g/L LYZ. The test results for the PEDOT-modified SPE are shown in **Figure S8(b)**. Similar to the Bare-SPE, the PEDOT-modified SPE's DPV peak value decreased to 24.45% of its original value after soaking in 10 g/L BSA and to 18.37% of its original value after soaking in 5 g/L LYZ, indicating that the protein molecules adsorbed on the surface severely affected the electron transfer capability of the electrode surface, resulting in a decrease in current value. Finally, the test results for the PEDOT-EG_4-OMe-modified SPE are shown in **Figure S8(c)**. After soaking in 10 g/L BSA, the DPV peak value of this electrode retained 96.29% of its original value, and after soaking in 5 g/L LYZ, it maintained 84.18% of its original value. This indicates that after rinsing, only a few protein molecules remained on the electrode surface, causing minimal impact on the electron transfer capability of the electrode surface. Therefore, the DPV peak values were close to the original values, demonstrating the excellent antifouling properties of the PEDOT-EG_4-OMe-modified SPE.

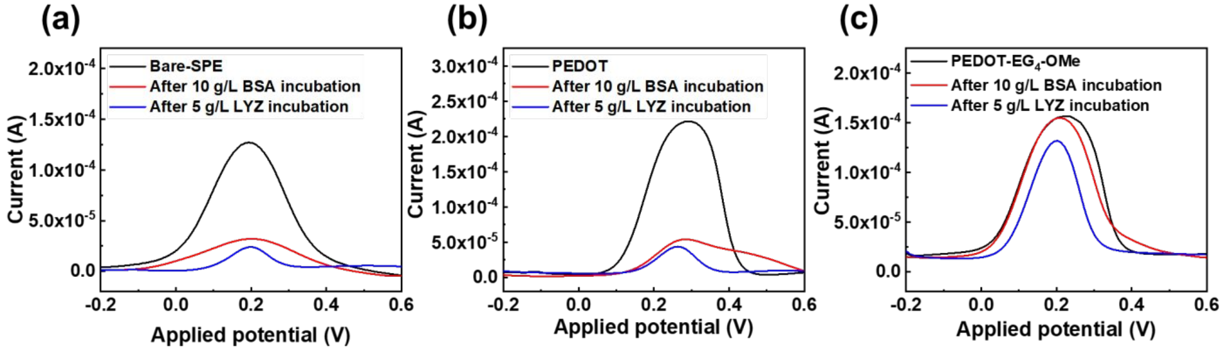

Figure S8. DPV peak value changes before and after soaking in 10 g/L BSA or 5 g/L LYZ solutions of (a) Bare-SPE. (b) PEDOT-modified SPE. (c) PEDOT-EG_4-OMe-modified SPE.

Calculation of detection limits

To calculate the detection limit of dopamine sensing, we first plot the normalized ECL intensity (y-axis) against the logarithm of dopamine concentration (x-axis). Next, we perform a linear fit to obtain an equation describing the relationship between ECL intensity and dopamine concentration. The standard error of the intercept from the fit is considered as noise (N). Finally, using the standard signal-to-noise ratio for detection limits (S/N = 3), the detection limit is calculated as the dopamine concentration at which the normalized ECL intensity reaches 1 - 3N. This concentration represents the detection limit.

Dopamine detection in the presence of both macromolecular and small molecule interferences

To evaluate the dopamine sensing capability of PEDOT-EG_4-OMe-modified SPE in the presence of both small molecule and macromolecule interferences, we conducted experiments to detect DA molecules in a mixture of MEM solution with 0.5 g/L BSA and LYZ proteins. As shown in **Figure S9**, the ECL emission of $Ru(bpy)_3^{2+}$/TEA on the PEDOT-EG_4-OMe-modified SPE exhibited an excellent linear relationship between ECL intensity and DA concentration after logarithmic transformation in the ranges of 5 nM to 250 µM and 250 µM to 2.5 mM. The limit of detection for the PEDOT-EG_4-OMe-modified SPE was estimated to be 2.015 nM. These detection limits are nearly the same as those achieved when detecting DA molecules without interferences. This indicates that the PEDOT-EG_4-OMe polymer films have high resistance to both small molecule and macromolecule interferences.

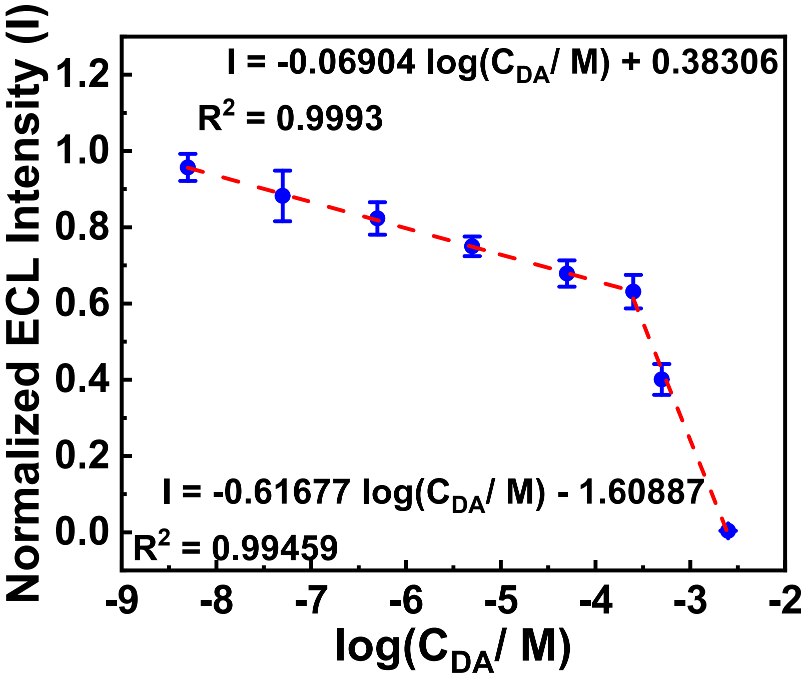

Figure S9. Calibration plot for DA detection in MEM solution with 0.5 g/L BSA and LYZ on PEDOT-EG_4-OMe-modified SPE.

Wavelength vs intensity plot

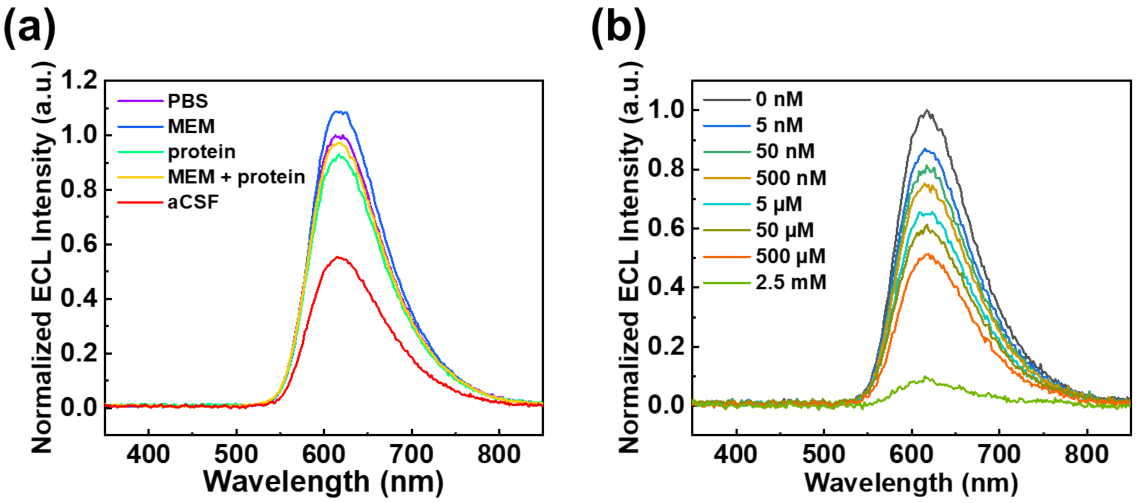

Figure S10. Wavelength vs. intensity plots for the ECL test of PEDOT-EG4-OMe-modified SPE: (a) in various interference solutions, (b) with different dopamine concentrations in aCSF.

Comparison of ECL dopamine sensing ability in real samples with other work

We aim to compare the linear range and limit of detection (LOD) of the ECL sensing platform for dopamine detection. As shown in **Table S1**, we compare the LOD for the ECL system based on the same luminophore, $Ru(bpy)_3^{2+}$, with different electrodes. Additionally, as illustrated in **Table S2**, we compare the LOD of dopamine sensors used in different biological samples, including dopamine hydrochloride injection, human urine, diluted whole blood, and artificial cerebrospinal fluid used in this study. The detection limits for these applications range from 1.7 nM to 6.59 μM. For simpler biological samples, such as dopamine hydrochloride injection, the detection limit is lower, whereas for more complex samples, like whole blood, the detection limit is higher. This demonstrates the excellent detection capability of the proposed ECL sensor, along with our work, for dopamine in complex biological samples.

Table S1. Comparison of the $Ru(bpy)_3^{2+}$-ECL sensing platform for DA with other works.

Luminophore	Linear range	LOD	Electrode	Reference
**$Ru(bpy)_3^{2+}$**	200 nM - 50 μM	85 nM	Pt electrode	1
**$Ru(bpy)_3^{2+}$**	10 nM - 600 μM	4 nM	GCE	2
**$Ru(bpy)_3^{2+}$**	10 nM - 10 μM	1.0 nM	GCE	3
**$Ru(bpy)_3^{2+}$**	1 μM - 50 μM	500 nM	SPE	4
**$Ru(bpy)_3^{2+}$**	1 nM - 200 μM	**0.887 nM**	SPE	**This work**

Table S2. Comparison of the ECL sensing platform for DA in different analysis sample.

Luminophore	Linear range	LOD	Analysis sample	Reference
**AuNPs**	9 - 200 μM	6.59 μM	Goat serum / artificial urine (100-folds diluted)	5
**CdSeTe/ZnS QDs**	0.375 - 450 μM	100 nM	Whole blood (10-folds diluted)	6
**PTh-D**	0.1 - 50.0 μM	40 nM	Human urine	7
**$Ru(bpy)_3^{2+}$**	5 nM - 500 μM	1.7 nM	Dopamine hydrochloride injection	8
**$Ru(bpy)_3^{2+}$**	1 nM - 200 μM	**3.119 nM**	Artificial cerebrospinal fluid / protein solution	**This work**

References

(1) Kong, D. X.; Liu, Y. J.; Wang, Z. M.; Jiang, L. L.; Cheng, J. S.; Xu, L. P.; Zhuang, Q. Z.; Lu, C. H.; Chi, Y. W.; Wei, Q. H. Establishment of an electrochemiluminescence quenching method for ribavirin detection in $Ru(bpy)_3^{2+}$ /TEA system. *J. Electroanal. Chem.* **2017**, *801*, 1-6.

(2) Li, X. Y.; Du, X. Z. Surface enhanced electrochemiluminescence of the $Ru(bpy)_3^{2+}$/ tripropylamine system by $Au@SiO_2$ nanoparticles for highly sensitive and selective detection of dopamine. *Microchem. J.* **2022**, *176*.

(3) Saqib, M.; Bashir, S.; Ali, S.; Hao, R. Highly selective and sensitive detection of mercury (II) and dopamine based on the efficient electrochemiluminescence of $Ru(bpy)_3^{2+}$ with acridine orange as a coreactant. *J. Electroanal. Chem.* **2022**, *906*.

(4) Kwon, H. J.; Rivera, E. C.; Neto, M. R. C.; Marsh, D.; Swerdlow, J. J.; Summerscales, R. L.; Uppala, P. P. T. Development of smartphone-based ECL sensor for dopamine detection: Practical approaches. *Results in Chemistry* **2020**, *2*.

(5) Rao, Z. K.; Guo, B. Y.; Zu, J. X.; Zheng, W. Q.; Xu, Y.; Yang, Y. T. Construction of an ECL-DPV Dual Model Biosensor for Dopamine Detection Based on PSO-ANN Algorithm. *IEEE Sens. J.* **2024**, *24* (6), 7463-7472.

(6) Stewart, A. J.; Hendry, J.; Dennany, L. Whole Blood Electrochemiluminescent Detection of Dopamine. *Anal. Chem.* **2015**, *87* (23), 11847-11853.

(7) Li, J. X.; Li, X. J.; Zhang, Y. H.; Li, R. X.; Wu, D.; Du, B.; Zhang, Y.; Ma, H. M.; Wei, Q. Electrochemiluminescence sensor based on cationic polythiophene derivative and NH_2-graphene for dopamine detection. *Rsc Advances* **2015**, *5* (7), 5432-5437.

(8) Wu, B. N.; Miao, C. C.; Yu, L. L.; Wang, Z. Y.; Huang, C. S.; Jia, N. Q. Sensitive electrochemiluminescence sensor based on ordered mesoporous carbon composite film for dopamine. *Sensors and Actuators B-Chemical* **2014**, *195*, 22-27.
